# Large scale genome skimming from herbarium material for accurate plant identification and phylogenomics

**DOI:** 10.1186/s13007-019-0534-5

**Published:** 2020-01-04

**Authors:** Paul G. Nevill, Xiao Zhong, Julian Tonti-Filippini, Margaret Byrne, Michael Hislop, Kevin Thiele, Stephen van Leeuwen, Laura M. Boykin, Ian Small

**Affiliations:** 1 Australian Research Council Centre for Mine Site Restoration, School of Molecular and Life Sciences, Curtin University, GPO Box U1987, Perth, WA 6102 Australia; 2 School of Biological Sciences, The University of Western Australia, Crawley, WA 6009 Australia; 3 Kings Park and Botanic Garden, Fraser Ave, Kings Park, WA 6005 Australia; 4 Australian Research Council Centre of Excellence in Plant Energy Biology, The University of Western Australia, Crawley, WA 6009 Australia; 5 School of Molecular Sciences, The University of Western Australia, Crawley, WA 6009 Australia; 6 Biodiversity and Conservation Science, Department of Biodiversity, Conservation and Attractions, Locked Bag 104, Bentley Delivery Centre, Bentley, WA 6983 Australia; 7 School of Molecular and Life Sciences, Curtin University, GPO Box U1987, Perth, WA 6102 Australia

**Keywords:** Chloroplast, Genome skimming, Herbarium specimens, Next-generation sequencing, Pilbara, Plant DNA barcoding, Plastid genome

## Abstract

**Background:**

Herbaria are valuable sources of extensive curated plant material that are now accessible to genetic studies because of advances in high-throughput, next-generation sequencing methods. As an applied assessment of large-scale recovery of plastid and ribosomal genome sequences from herbarium material for plant identification and phylogenomics, we sequenced 672 samples covering 21 families, 142 genera and 530 named and proposed named species. We explored the impact of parameters such as sample age, DNA concentration and quality, read depth and fragment length on plastid assembly error. We also tested the efficacy of DNA sequence information for identifying plant samples using 45 specimens recently collected in the Pilbara.

**Results:**

Genome skimming was effective at producing genomic information at large scale. Substantial sequence information on the chloroplast genome was obtained from 96.1% of samples, and complete or near-complete sequences of the nuclear ribosomal RNA gene repeat were obtained from 93.3% of samples. We were able to extract sequences for the core DNA barcode regions *rbcL* and *matK* from 96 to 93.3% of samples, respectively. Read quality and DNA fragment length had significant effects on sequencing outcomes and error correction of reads proved essential. Assembly problems were specific to certain taxa with low GC and high repeat content (*Goodenia*, *Scaevola*, *Cyperus*, *Bulbostylis*, *Fimbristylis*) suggesting biological rather than technical explanations. The structure of related genomes was needed to guide the assembly of repeats that exceeded the read length. DNA-based matching proved highly effective and showed that the efficacy for species identification declined in the order cpDNA >> rDNA > *matK* >> *rbcL.*

**Conclusions:**

We showed that a large-scale approach to genome sequencing using herbarium specimens produces high-quality complete cpDNA and rDNA sequences as a source of data for DNA barcoding and phylogenomics.

## Background

Herbaria are valuable sources of curated plant specimens that are often linked to extensive metadata. They have been described as “treasure troves” [1] of information and are increasingly the focus of tissue samples for DNA barcoding and phylogenetic studies, where specimens with accurate taxonomic identification and associated metadata are essential (e.g. [2–4]). Their use as sources of DNA is particularly important when the target species are distant, found in isolated or hard to access locations, are difficult to identify, or when studies are at large scales [2].

Herbaria are now accessible to genetic studies because of advances in high-throughput, next-generation sequencing (NGS) methods. The genome-skimming approach, where highly repetitive genome regions such as rDNA and organelle genomes are recovered using shallow-pass genome sequencing [5], has been used to retrieve plastid DNA and rDNA sequences from 146 herbarium specimens [6], to sequence the nuclear genome of a *Arabidopsis thaliana* herbarium specimen [1], to improve phylogenetic resolution in Acacia [4], and recover rDNA and plastid genome sequences from 25 herbarium specimens up to 80 years old from 16 different Angiosperm families [7]. However, large scale studies with broad taxonomic sampling are lacking but needed given the future importance of herbaria for the systematic development of reference barcode databases [2].

This project used recent developments in full genome sequencing to provide a DNA sequence database of a key set of the Pilbara flora, and provides a proof of concept as an initial stage in the development of effective large scale, DNA-based species identification system for the Pilbara bioregion. The Pilbara bioregion of Western Australia is an area of national importance as it is rich in biodiversity [8] and is one of 15 national biodiversity hotspots [9]. The region is also of international importance as it is a major global producer of iron ore and lithium [10]. Effective identification of plant species is critical for conserving the rich and diverse flora of the Pilbara bioregion, particularly in the context of the challenges presented by resource development associated with mining. Environmental impact assessment and native vegetation clearing approval processes require certainty in the identification of species, yet this can be extremely challenging in such a vast, remote and climatically episodic region as the Pilbara. Development of an improved knowledge base for the Pilbara flora will deliver improved reliability and efficiency of plant identifications for environmental impact assessments and associated regulatory land use planning approval processes.

As an applied assessment of the large-scale recovery of plastid and ribosomal genome sequence from herbarium material using a genome-skimming approach, we sequenced 672 samples covering 21 families, 142 genera and 530 named and proposed named species (i.e. species with manuscript or phrase names). Our aim was to assess whether the successes of previous studies using this approach could be repeated with a large number of species from many plant families. First, we identified the proportion of species in families for which complete or near complete plastid genome, rDNA, *matK* and *rbcL* were retrieved in the sequencing dataset. We then explored the impacts of various parameters (e.g. DNA fragment size, number of raw reads, depth of mapped reads, DNA quantity and quality) on assembly error. Finally, as a proof of concept, we tested the efficacy of the DNA sequence information for identifying plant samples using 45 specimens recently collected in the Pilbara, and discuss current and potential future uses of the data.

## Results

**Fig. 1:**
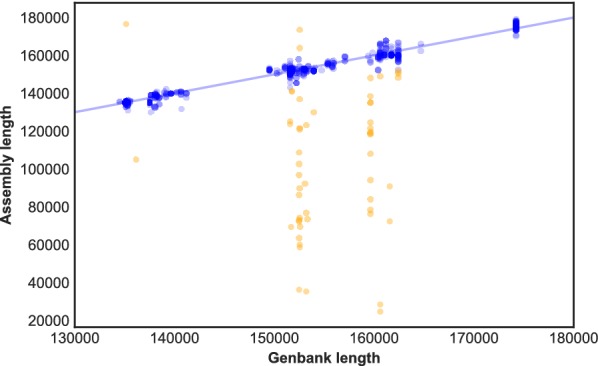
Estimation of assembly completeness by comparison with Genbank records. Assemblies were paired with the closest match amongst all complete plastid genomes in Genbank. The scatter plot shows the relationship between the length of the assembly and its paired Genbank record. The straight line indicates the expected (x = y) values. The colours indicate ‘good’ (blue) and ‘poor’ (orange) assemblies based on the discrepancy observed between the paired lengths (calculated as described in the Methods). In all, from 672 samples, 606 assemblies passed this criterion, 54 assemblies failed, and for 12 samples no assembly was obtained

**Fig. 2:**
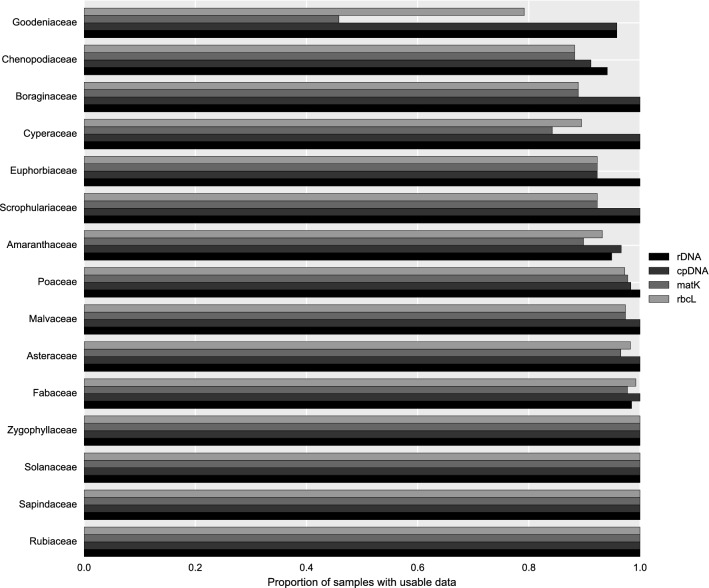
Proportion of species in families for which complete or near complete plastid genome, rDNA, *matK* and *rbcL* were retrieved in the sequencing dataset. Families shown are those with more than five species in the study

**Fig. 3:**
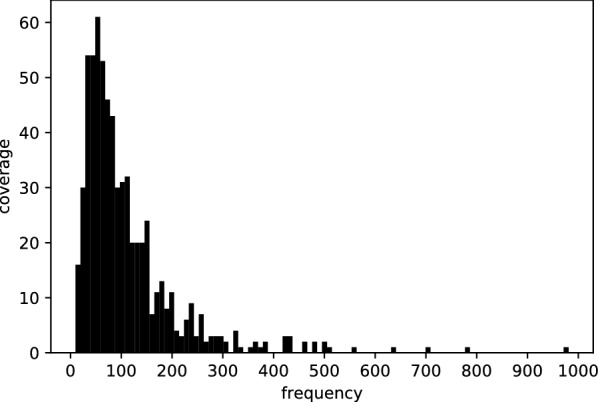
The distribution of coverage across all the samples

Between 1,800,158 and 10,692,690 high-quality paired-end reads were produced from each sample (average 4,922,683; median 4,960,988). Sequence assembly was attempted for 672 samples, covering 530 named or proposed species. Complete or near complete sequence information on the chloroplast genome was obtained for 96.1% of samples, and complete or near-complete sequence of the nuclear rDNA repeat for 93.3% of samples (Figs. 1 and 2). The remaining samples were of too poor quality for successful assembly. Most samples gave around 30–150× coverage of the chloroplast genome (Fig. 3). We were able to extract sequences for the core DNA barcode regions (*rbcL* and *matK*) from 96.4% and 93.3% of samples, respectively (Fig. 1).

**Fig. 4:**
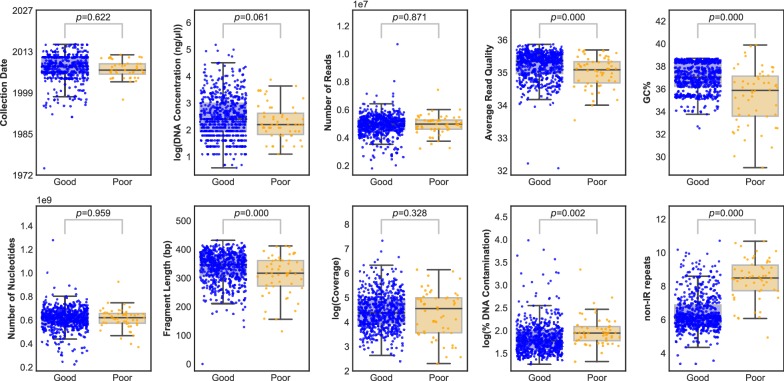
Relationships between various DNA, sequencing and assembly parameters on assembly completeness**.** The distributions of ten different parameters that might influence assembly success were investigated in samples that were deemed to be ‘good’ (blue) or ‘poor’ (orange) (as described in Methods and depicted in Fig. 1). Individual points represent individual samples; box plots indicate the median (centre line), interquartile range (box) and 1.5× interquartile range (‘whiskers’). The *p*-values shown indicate the results of t-tests for differences in the means of the two distributions in each case

The yields of DNA were generally low, ranging from 10 ng to 2 µg, but sufficient for the task as the quantity of DNA did not affect assembly quality (Fig. 4). Specimen age had no effect on assembly error either, but the DNA from some samples was highly fragmented and DNA fragment length was significantly correlated with assembly outcomes (Fig. 4). We tested the effect of seven other parameters on assembly error. Neither the number of raw reads for each sample, the number of nucleotides, nor the depth of mapped reads (Fig. 4) correlated with assembly error, confirming that reads weren't limiting; however, read quality and DNA contamination had a significant effect (Fig. 4). Two biological parameters, GC content and repeat content, were strongly associated with assembly success (Fig. 4).

### Proof of concept

The efficacy of DNA sequence information for identifying plant samples was tested using 45 specimens recently collected in the Pilbara as part of the Ausplots Rangelands survey project [11]. These specimens were selected to represent samples with morphological identifications that corresponded to species already in the database and we focused on difficult to identify grasses. Sequencing and assembly of rDNA and cpDNA sequences was done using the same approach as other samples. The average common substring method [12] was used to match the rDNA and cpDNA sequences to the database of Pilbara samples. Species identification for these specimens was also attempted using the short barcode sequences from the *rbcL* and *matK* genes. The *rbcL* and *matK* barcode sequences were extracted from the assembled cpDNA sequences by simulated PCR [13] using combinations of primers obtained from Barcode of Life Datasystem (BOLD). Extracted barcodes were used to search the PILBseq database as described above.

DNA-based matching of the 45 ‘known unknown’ specimens agreed with the morphology-based identification at the genus level in every case for both rDNA and total chloroplast DNA (cpDNA), and in almost every case when using specific chloroplast barcode regions such as *rbcL* or *matK*. DNA-based matching agreed with the morphology-based identification at species level ~ 70% of the time for rDNA sequences and 83% of the time for cpDNA sequences. DNA-based matching showed that matching effectiveness decreased in the following order, cpDNA >> rDNA > *matK* >> *rbcL*.

## Discussion

We demonstrated that a large scale approach to genome sequencing of herbarium specimens can produce a large dataset of complete cpDNA and rDNA sequences, and that the data generated can be used for species identification and phylogenomics. Our study included a broad range of families and genera and DNA was of varying concentration and quality. Our success is important and demonstrates that herbaria can be used as a source of plant material for building a comprehensive DNA barcoding and metabarcoding database.

### Lessons learned

We have learned a number of valuable lessons from this study and future projects will greatly benefit from this new knowledge. Before DNA was extracted, experienced botanists at the Western Australian Herbarium checked and confirmed identifications. This proved to be a critical step in the project as it revealed misidentifications, provided more complete identifications (e.g. to infra-species) in some cases, and resulted in the exclusion of some specimens that could not be identified accurately or had a complex, unresolved taxonomy.

Complete or near complete sequence information on the chloroplast genome and the nuclear rDNA was obtained for a high proportion of samples. Most samples gave coverage of the chloroplast genome sufficient for high-quality assembly. Raising coverage by multiplexing fewer samples would increase the proportion of complete genomes but reduce the total number of genomes obtained, so we believe that the level of multiplexing chosen maximised the cost-effectiveness of the project. Where reads were limiting for full de novo assembly, assemblies were constructed by aligning contigs and reads to a closely related reference genome. In these cases, despite the care taken to ensure consistency between the assembly and the input reads, there is a low risk that the gene order in the assembly is not correct if the true order differs from that in the reference used. The rare assembly failures were due either due to sub-standard DNA sequence quality/quantity or biological peculiarities specific to certain taxa (notably *Goodenia*, *Scaevola*, *Cyperus*, *Bulbostylis*, *Fimbristylis*). In general, genomes from these problematic genera contained extensive low-GC intergenic regions including many repeats that made assembly with this short-read data difficult or impossible. Future studies of this type aimed at such taxa will need to include data from long-read sequencing technologies to eliminate these issues.

DNA extraction can prove problematic when using herbarium material; however reliable extraction of DNA and recovery of sequence data from samples of various ages is possible [14, 15]. In our study, even though DNA was degraded and yields of DNA were generally low, in most cases they were adequate for all downstream molecular techniques required for the project. Short read sequencing deals with short fragments and abasic sites quite well [16], but error correction of the reads before assembly proved essential. Repeats that exceeded the read length led to problems with unambiguous assembly of contigs but we overcame this limitation by using the structure of related genomes to guide assembly. Finally, we found significant differences in chloroplast DNA proportions, with aphyllous plants (e.g. *Tecticornia*) appearing to have less chloroplast DNA, which led to limited coverage and made it more difficult to assemble reads.

### Proof of concept

We tested the efficacy of the DNA sequence database by sequencing 45 new samples supposedly corresponding to species already in the database and treated them as ‘known unknowns’ in analyses. These samples were mostly hard-to-identify grasses. In the 7 cases that the cpDNA match disagreed with the morphology-based identification, the rDNA match also disagreed, and in 5 of the 7 cases the rDNA match was to the same species as the cpDNA match. This suggests that 5 of the 7 apparent 'errors' in the cpDNA matches (and quite possibly all 7) are due to misidentification of either the 'known unknown' or the original database sample, or due to taxonomy errors (i.e. taxonomic species boundaries incongruent with actual genetic relationships). At the species level, DNA-based matching showed that the efficacy for species identification declined in the order cpDNA >> rDNA > *matK* >> *rbcL*. This is consistent with the findings of other studies [17].

cpDNA genomes from this study were invaluable in helping resolve the phylogenetic backbone of another important Pilbara genus, *Ptilotus* (Amaranthaceae) [18]. A phylogeny of selected species based on the genome sequences from this study had very high support for most nodes. Applying this phylogeny as a topological constraint on a larger (more species-complete) phylogeny based on Sanger sequencing of a limited set of markers provided substantially improved backbone resolution and support. Finally, cpDNA genomes from this study have also been combined with existing chloroplast genomic sequences to examine the diversification timing of an Australian arid zone grass species complex (*Tridoia basedowii*) [19].

### Re-use potential

We plan to use these data in a molecular identification system for Western Australian flora. This will enable identification of specimens throughout the year (e.g. non-flowering times) and for morphological hard-to-identify species (e.g. those with constrained or reduced morphological characters). It will also have practical applications in a wide range of ecological contexts, such as gut and scat analysis of animals to determine dietary preferences of threatened (e.g. [20]), and checking the integrity of seed collections for seed banking and use in land restoration/revegetation programs [21]. The availability of this technology will modernize plant surveys by reducing constraints on survey effort through moderating sampling timing restrictions and seasonal effects and enabling rapid identification and assessment of regional context. The technology will also facilitate greater certainty for environmental impact assessments and associated land using planning processes. However, there are many other potential uses of extensive plastid sequence data beyond species identification [22]. Like the *Ptilotus* [18] and *Triodia* [19] studies, the sequences from this project could be used to improve the resolution of plant phylogenies, which are increasingly based on the integration of samples, some with short marker sequences and others complete genomes. A third potential use of the data is studies of the evolution of plastid genome function, including understanding adaptive changes (e.g. [23, 24]).

## Conclusions

In this study, we have shown that we can readily produce at scale, whole chloroplast and ITS rDNA data from herbarium specimens that can be used for a range of applications. The project represents the first extensive collection of whole plastid genome data in Australia. The data are open access and available on several databases (our data portal and the SRA) for use by environmental consultants, researchers and government agencies. We envisage that this will be a ‘living’ dataset, in that the sequence coverage will continue to grow as samples are added, new ways to analyse and use the data are developed, other environmental datasets are linked and new users contribute to the resource.

This project represents a proof of concept and a first step in the development of a molecular identification system for the Pilbara flora. To be fully effective, the database needs to be representative of all species present in the Pilbara bioregion. However, the current database covers the majority of two major families (Poaceae and Asteraceae) where taxonomic identification can be challenging, and so has current application for molecular identification in these families.

Priorities for future work include incorporating additional species for taxon completeness in the Pilbara bioregion, and including multiple samples per species. The challenges and limitations of biodiversity surveys and assessments (i.e. traditional taxonomic identification and field observation) are familiar to those responsible for environmental stewardship. This dataset provides an exceptional opportunity to evaluate the utility of a molecular approach for accurate, timely and cost-effective species identification that is critical for effective biodiversity management, sustainable use and restoration monitoring.

## Methods

### Species selection

Species were selected in consultation with taxonomic and identification experts at the Western Australian Herbarium, based on the following criteria: (1) Conservation-priority species that occur on mining tenure in the Pilbara and are sometimes difficult to identify because they are character-poor or often sterile or poorly known; (2) For each represented family, all other species that occur on mining tenure in the Pilbara; and (3) Additional off-tenure species that increased the completeness of coverage for families, genera and/or species complexes in the Pilbara. Samples for sequencing were taken from specimens lodged at the Western Australian Herbarium (PERTH). Specimens suitable for sequencing were selected according to the following criteria: (1) Collected in the last 10 years (with some exceptions due to a lack of more recent specimens); (2) Sufficient material on the specimen, so as to not compromise future use for other purposes; (3) Collected from the Pilbara bioregion (with some exceptions due to a lack of more recent specimens); (4) Well-dried and of suitable quality for reliable identification.

Identifications of all sampled specimens were confirmed by botanists at the Western Australian Herbarium. This was an important step, to reduce as much as possible the likelihood of sequences bearing an incorrect initial identification. Some selected specimens were changed to avoid potential problems or where this confirmation step showed that the specimen was mis-identified. Contextual data from herbarium records, including sampling location, site descriptions, and associated vegetation, were linked and recorded for each specimen and are found on the project data portal (https://pilbseq.dbca.wa.gov.au/).

### DNA extraction

DNA was extracted from herbarium samples using the commercial DNeasy Plant Mini Kit (Qiagen) following the manufacturer’s instructions. DNA was eluted in 100 μl of AE buffer and DNA concentration and quality was quantified on a NanoDrop ND-1000 spectrophotometer (ND-1000; Thermo Fisher Scientific), with confirmation through gel electrophoresis and QUBIT fluorometric quantitation for a subset of samples. Minimum concentration for sequencing was 1 ng/ul. Samples were sequenced at the AGRF node in Melbourne, Victoria. Where required, DNA samples were purified and concentrated using a DNA Clean & Concentrator™-5 Kit (Zymo Research).

### DNA sequencing

Even though DNA samples were generally of low molecular weight, DNA from all samples was sheared in a volume of 50 µl using a Covaris E220 Focused Ultrasonicator. Following shearing, sequencing libraries were prepared using Illumina’s TruSeq Nano DNA Library preparation kit (350 bp median insert) following the manufacturer’s protocol. Pilot sequencing showed that Truseq libraries provided more even genome coverage than transposon-tagged libraries. Libraries were assessed by gel electrophoresis (Agilent D1000 ScreenTape Assay) and quantified by qPCR (KAPA Library Quantification Kits for Illumina). Sequencing was performed on the Illumina HiSeq 2500 system with 2 × 125 nt paired end reads using the HiSeq PE Cluster Kit, v5 and HiSeq SBS Kit, v4 (250 cycles).

### Sequence processing

To test the suitability of the data for future uses including the development of a molecular identification service and phylogenomic studies, draft plastid genome assemblies were undertaken for the complete dataset using the following workflow (see also Additional file 2). We first removed adapter sequences with cutadapt (v1.9.1) [25]. We then normalized read depth based on k-mer counts using BBNorm, (a tool in the BBMap package), with a k-mer low/high coverage cut-off of 10/500 [26]. Read errors were corrected using SPAdes (v3.6.1) [27] and overlapping paired-end reads were merged using BBMerge (v8.82), another tool in the BBMap package. Merged reads were assembled with Velvet (v1.2.10) [28] with k-mer values of 51, 71, 91 and 111, and with low coverage cut-off values of 10, 7, 15 and 20. Velvet assembly was terminated if assembled contigs of a plastid under one set of parameters met the desired criteria for length and k-mer coverage. Assembled contigs were aligned with all sequences in a local database comprising plastid genomes from GenBank (1366 organisms) and the newly assembled genomes in this study using MUMmer (version 3.23) [12]. For each assembly, the best reference sequence was chosen as that which covered the most sequence with the least number of contigs based in the alignment. Aligned contigs were then ordered, oriented and connected directly to be a single longer sequence if the assembled plastid sequences satisfied the above requirement. Otherwise, Velvet assembly under other coverage cut-off and k-mer values was launched until all running rounds for one sample were finished. When quality contigs were not yet obtained using the de novo approach, assembly was switched to reference-guided approach. The best pair of a reference and contigs set in all alignments was chosen and plastid contigs were connected as a single sequence. Additionally, two seed-extension assemblers, The ORGanelle ASeMbler (v b2.2) [29] and NOVOPlasty (v.2.5.9) [30] were tested and their assemblies retained in 242 and 35 cases, respectively. Finally, draft genomes were refined by filling gaps using GapFiller (v1.10) [31], mapping the raw reads to the genomes using BWA (v0.7.5a-r405) [32] and correcting and verifying the assembly with Pilon (v1.16) [33].

Assembly errors were estimated by comparing the assembly length to the length of the most similar complete chloroplast genome in Genbank. Genome matches were ranked by the average common substring method [12]. Error was calculated as abs(log(assembly length/Genbank match length)). An assembly with an error of 0.1 or greater was considered as a poor assembly (equivalent to a length discrepancy of ~  ± 10%, with missing sequence counting more towards the error than duplicated or extra sequence). This is only a rough guide to assembly quality as in many cases no closely related genomes were available for comparison. Statistical tests (t-tests) were performed to evaluate the impact of parameters such as sample age, DNA concentration, number of raw reads, fragment length, read coverage, GC content or repeat content on assembly error. Repeats were analysed using Vmatch (https://www.vmatch.de), based on REPuter [34]. The lengths of non-IR repeats were summed to give a single value per assembly.

See Additional files 1 and 2 for summaries of the bioinformatics and entire project workflows.

## Supplementary information

**Additional file 1.** Workflow summarising DNA sequence assembly.

**Additional file 2.** Workflow summarising the methodological approach employed in this study to produce a DNA sequence resource.

## Data Availability

The data set supporting the results of this article is available at the PILBseq project data portal (https:/pilbseq.dbca.wa.gov.au/) as well as in the SRA [PRJNA522689]. The publicly available PILBseq portal has been developed to facilitate access to raw data (FASTQ) and the metadata associated with each accession (https:/pilbseq.dbca.wa.gov.au/). In our portal, each sample is linked to extensive metadata (e.g. species description, conservation status, distribution maps) collated by Western Australia’s biodiversity and conservation agency. The website also has a facility to search for the most similar sequence in the database.

## Abbreviations

AGRF: Australian Genome Research Facility
SRA: sequence read archive
BGPA: Botanic Gardens and Parks Authority
BPA: Bioplatforms Australia
UWA: the University of Western Australia
