# Supplementary figures and images for: Large scale genome skimming from herbarium material for accurate plant identification and phylogenomics
Source: Plant Methods. 2020 Jan 4;16:1. doi: 10.1186/s13007-019-0534-5 (PMC6942304; doi:10.1186/s13007-019-0534-5)

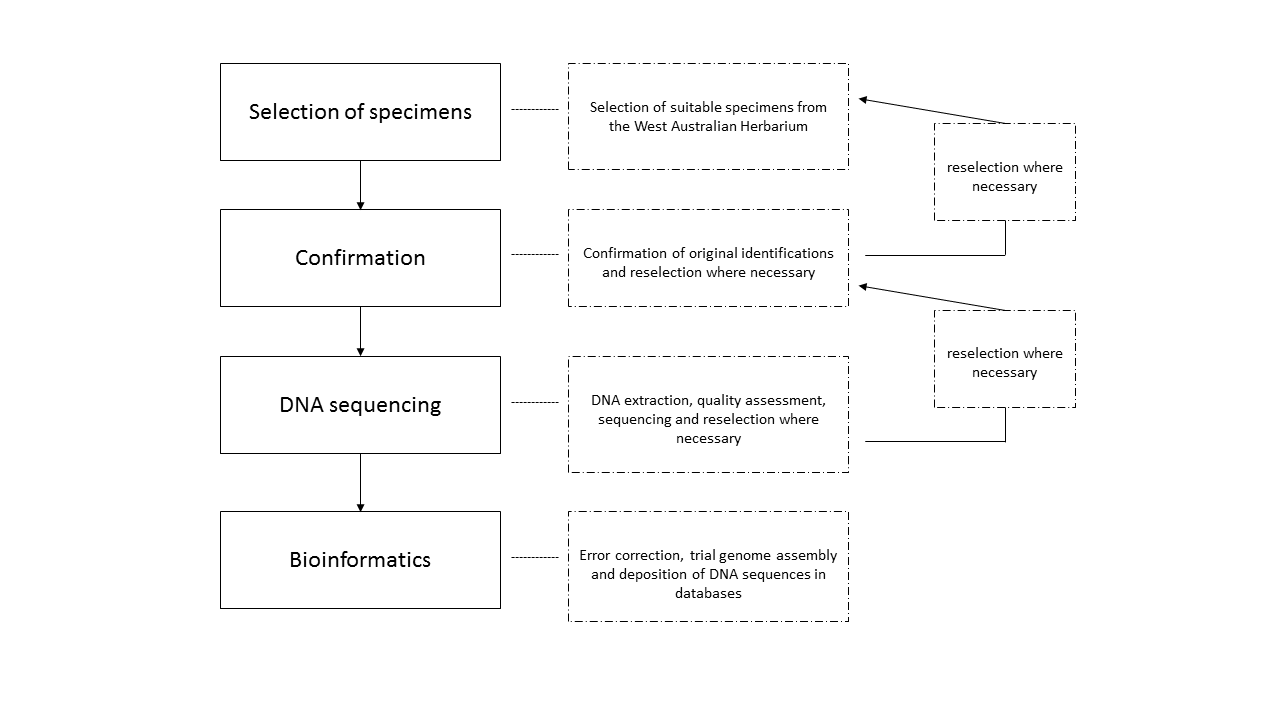

Supplement: Supplementary file 1 — Additional file 1. Workflow summarising DNA sequence assembly. [file 13007_2019_534_MOESM1_ESM.docx]

**
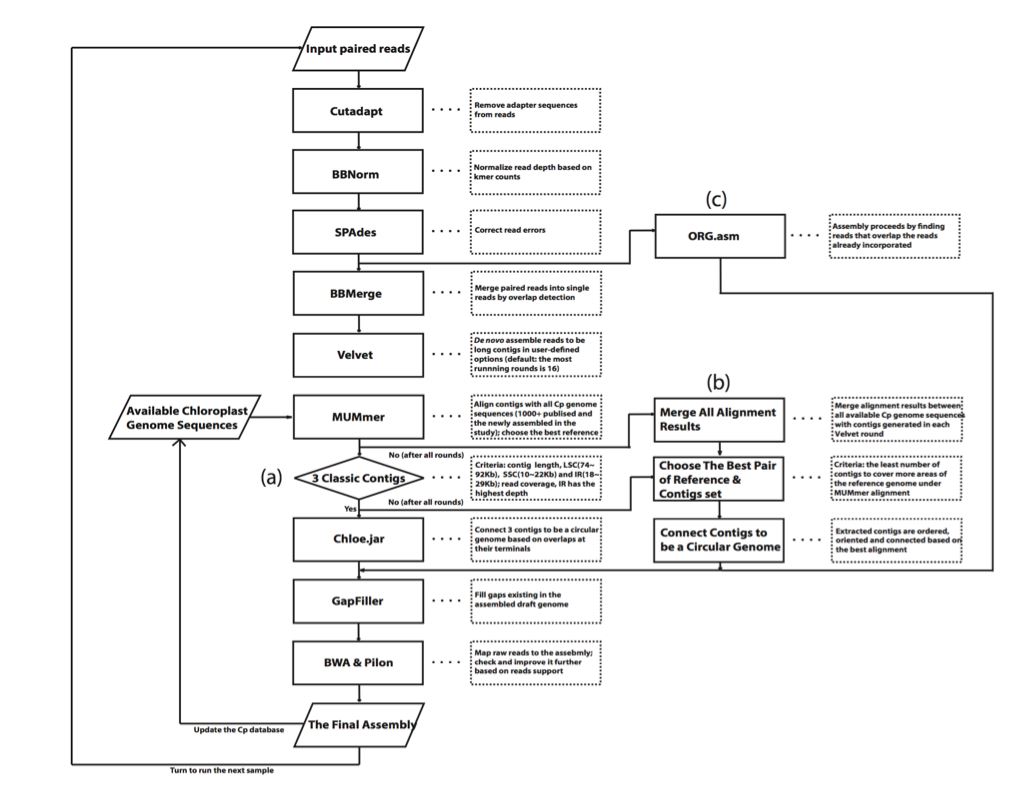
**

Supplement: Supplementary file 2 — Additional file 2. Workflow summarising the methodological approach employed in this study to produce a DNA sequence resource. [file 13007_2019_534_MOESM2_ESM.docx]
